# Study on Quality of Life of Patients with Obstructive Sleep Apnea—Pilot Study

**DOI:** 10.3390/medicina61071234

**Published:** 2025-07-08

**Authors:** Olja Tanellari, Brunilda Koci, Edlira Baruti Papa, Carina Balcos, Adina Oana Armencia, Tinela Panaite, Irina Zetu

**Affiliations:** 1 Surgery Department, Faculty of Dental Medicine, “Grigore T. Popa” University of Medicine and Pharmacy, 700115 Iasi, Romania; oli_koca@yahoo.com (O.T.); carina.balcos@umfiasi.ro (C.B.); tinela-panaite@umfiasi.ro (T.P.); nicoletazetu@gmail.com (I.Z.); 2 Department of Stomatology, Faculty of Dental Sciences, Aldent University, 1005 Tirane, Albania; brunilda.koci@ual.edu.al; 3 Department of Dentistry and Maxillofacial Surgery, Salus Hospital, 1005 Tirana, Albania; andiaaura@gmail.com

**Keywords:** sleep apnea, obstructive, quality of life, questionnaires, sleep disorders

## Abstract

*Background and Objectives*: Sleep apnea (OSA) significantly impacts patients’ health, affecting cognitive, emotional, and social functioning, thus reducing overall quality of life (QoL). Despite global research interest, there are limited data on the QoL of Romanian patients with OSA. *Aim of study*: This study aimed to assess the quality of life of patients diagnosed with OSA using the Sleep Apnea Quality of Life Index (SAQLI), a validated, disease-specific questionnaire. *Material and Methods*: A cross-sectional study was conducted between January 2021 and February 2022 on 28 adult patients from medical units in Iași (Romania) and Albania. The patients were clinically evaluated and confirmed to have OSA (AHI > 5). QoL was assessed pre-treatment using the SAQLI, covering daily activities, social interactions, emotional status, and symptomatology. Statistical analyses were performed using SPSS v26.0. *Results*: Most patients had moderate (32.1%) or severe OSA (53.6%). Lower QoL scores correlated with increased disease severity. Significant impairments were observed in daily functioning, social relationships, and emotional well-being. Patients with severe OSA reported the lowest scores across all domains. *Conclusions*: OSA severely affects quality of life, particularly in patients with moderate to severe forms. Early diagnosis and personalized, multidisciplinary management strategies are essential to improving outcomes and overall patient well-being.

## 1. Introduction

The quality of life (QoL) of individuals with obstructive sleep apnea syndrome (OSA) has garnered attention in the current literature due to its considerable prevalence and detrimental effects on the body. OSA is defined by the occurrence of repeated episodes of partial (hypopnea) or complete (apnea) cessation of breathing during sleep that last for a minimum of 10 s. In high-income countries, OSA affects 2% of middle-aged women and 4% of middle-aged men [1]. In overweight patients, the frequency of OSA may increase to 41% [2].

Obstructive sleep apnea (OSA) is increasingly recognized as a complex disorder with significant systemic implications, extending far beyond sleep-related respiratory disturbances. A growing body of literature has established strong associations between OSA and a range of chronic comorbidities, including cardiovascular conditions (such as hypertension, myocardial infarction, stroke, and arrhythmia), metabolic dysfunctions (notably insulin resistance and type 2 diabetes), and neurocognitive impairments, such as deficits in memory, attention, and executive function. These interconnected effects directly compromise functional capacity, occupational performance, and social engagement, ultimately diminishing overall quality of life. Given the multidimensional impact of OSA, the use of validated assessment instruments—particularly standardized questionnaires—has become indispensable for both clinical practice and research aimed at capturing the broader consequences of this condition [3,4,5].

The primary daytime symptoms of OSA are loud snoring and daytime sleepiness, as opposed to nocturnal symptoms [1]. The cardiovascular system is particularly affected by the general health consequences of the recurrent oxyhemoglobin desaturation and sudden awakenings that are characteristic of OSA [6]. This condition frequently leads to diagnoses such as pulmonary hypertension, resistant systemic hypertension, chronic heart failure, arrhythmia, myocardial infarction, stroke, and increased mortality.

All these consequences manifest in a reduced daily performance, decreased work efficiency, and deterioration in overall quality of life. Due to these complex effects, validated assessment tools—particularly in the form of questionnaires—have become essential in both the clinical evaluation of patients and in specialized research [7].

OSA also affects the nervous system by causing early-onset melancholy, irritability, cognitive disorders, concentration difficulties, short-term memory loss, and mood disorders [8]. These conditions are associated with OSA and have an impact on familial, professional, and social life. Additionally, they increase the likelihood of car and workplace accidents [9,10].

Consequently, OSA management, which entails the use of continuous positive airway pressure (CPAP), is regarded as the industry standard for enhancing neurocognition and daily performance, reducing daytime sleepiness, and increasing sleep quality [6,10,11].

Although obstructive sleep apnea (OSA) is well documented internationally, data on its impact on quality of life in Eastern Europe—particularly in Romania and Albania—are lacking. Cultural norms, lifestyle, and access to healthcare can influence how patients perceive and report symptoms, affecting scores obtained through standardized tools like the SAQLI. Without proper cultural adaptation, these instruments may reflect subjective perceptions rather than the actual severity of the condition. Moreover, the lack of specific guidelines for orthodontists, as noted by the American Association of Orthodontists, highlights existing gaps in the literature. Therefore, culturally validated assessment tools are essential for accurately understanding the impact of OSA and developing interventions tailored to local contexts [12].

Because of these multifaceted impacts, the use of standardized questionnaires has become essential for both patient assessment in clinical practice and for research purposes within the scientific community [7]. The quantity of instruments available for the early diagnosis of OSA has increased because of the rapid increase in interest in quality of life [9]. The Calgary Sleep Apnea Quality of Life Index (SAQLI), which was developed by Flemons and Reimer, appears to be the most comprehensive outcome measurement instrument, as it encompasses all four domains for measuring QoL [13].

Various domains have been the subject of numerous studies that have evaluated the quality of life of patients with obstructive sleep apnea. These studies include comparative studies between diagnosed patients and healthy subjects [14] and patients with OSA and healthy life partners [6], cross-sectional studies [10,11], and studies on CPAP-treated patients [6,15,16] with or without uvulopalatoplasty [17,18].

The concept of the quality of life of OSA patients is not fully understood, despite the fact that interest in this topic has increased in recent years. The treatment of OSA patients in a multimodal and holistic manner, which extends beyond medical treatment, is further supported by each new study conducted in this field. No quality-of-life studies had been conducted on OSA patients in Romania prior to our investigation. Consequently, the objective of this investigation was to assess the quality of life of patients with obstructive sleep apnea by employing the Sleep Apnea Quality of Life Questionnaire (SAQLI).

## 2. Materials and Methods

This cross-sectional study was conducted using quantitative research to investigate the perception of quality of life among patients with untreated OSA or those in the initial phase of treatment.

### 2.1. Patient Selection

The study group was composed of 28 patients who were recruited from Romania and Albania between January 2021 and February 2022, after obtaining approval from the Ethics Committee of the “Grigore T. Popa” University of Medicine and Pharmacy in Iași, Romania (Nr. 204/03 July 2022) and Aldent University Tirana, Albania (no. 31/01.06.2022). The medical team responsible for diagnosing and treating these patients had previously established a diagnosis of OSA. The small number of study participants were selected during the post-COVID-19 pandemic period, when patients presented in much smaller numbers for OSA diagnosis.

The study included subjects diagnosed with OSA who signed informed consent forms and presented nocturnal and daytime symptoms, as well as an apnea–hypopnea index (AHI) of at least five events per hour, diagnosed by polysomnography prior to the conduct of this study.

The inclusion criteria were as follows: adult subjects diagnosed with OSA who provided informed assent to participate, irrespective of their origin, religion, or ethnicity.

The following were considered exclusionary criteria: acute psychosis, suspected intellectual disability, diagnosed mental and cognitive disorders, and refusal to participate.

### 2.2. Quality of Life Measurement

The SAQLI questionnaire was validated and implemented to evaluate quality of life. Two authorized translators translated the questionnaire into Romanian, which was subsequently administered to each patient diagnosed with OSA [13]. Anthropometric, demographic, clinical, and medical history data were obtained from each participant.

The Sleep Apnea Quality of Life Questionnaire (SAQLI) is a questionnaire that has been specifically designed and validated for OSA, meeting the criteria for internal consistency, validity, and specificity. It emphasizes symptoms and a decline in adaptability to change. The SAQLI questionnaire is predominantly designed as an evaluation tool for measuring changes post-treatment, which allows it to reflect potential negative consequences of treatment.

The SAQLI comprises inquiries that are classified into the following five domains: (A) daily activity; (B) social interactions; (C) emotional status; (D) symptoms; and (E) treatment-related symptoms.

A 7-point Likert scale was employed to collect responses to the SAQLI questionnaire. The final questionnaire score was determined by summing the mean scores from the four domains and dividing the sum by four. Interpretation was facilitated by the fact that the total SAQLI score and each domain score ranged from 1 to 7. Domains A–D quantified the quality of life (QoL) results pre-treatment for OSA, while domain E was exclusively utilized for patients who were treated with CPAP [13,19]. The questionnaires have been validated for both populations [20,21].

### 2.3. Statistical Analysis

The SPSS 26.0 software (IBM, Armonk, New York, NY, USA) was employed to conduct statistical analyses. The data are presented as means, minimum and maximum values, and standard deviations. The distribution of data was assessed for normality using the Shapiro–Wilk test prior to parametric testing. The Pearson Chi-square test and ANOVA were employed to assess statistically significant differences between subjects categorized by OSA severity and responses. Following ANOVA, Bonferroni post hoc comparisons were used to identify inter-group differences, and multiple linear regression models were employed to assess the influence of AHI, BMI, age, and sex on QoL domains. A multiple linear regression analysis was performed to identify the association between quality-of-life scores (SAQLI domains) and clinical and demographic variables, including age, sex, body mass index (BMI), and apnea–hypopnea index (AHI). Statistical significance was defined as a *p*-value of less than 0.05.

## 3. Results

In the group of 28 participants diagnosed with OSA, the age ranged from 33 to 76 years, with an average of 56.54 ± 11.06 years (Table 1). Regarding the gender distribution, the female/male ratio was 11/17. Most of the subjects who participated in the study came from urban areas, with 75% of them being employed. In terms of apnea severity, 53.6% of the participants had a severe AHI (>30 events/hour), 32.1% had a moderate AHI (16–30 events/hour), and 14.3% had a mild AHI (<15 events/hour). Regarding body mass index, 42.9% of the participants were classified as obese (BMI > 30 kg/m^2^), 39.3% as overweight (BMI 28–30 kg/m^2^), and 17.9% had a normal-weight (BMI < 25 kg/m^2^) (Table 1).

The distribution of participants based on OSA degree reveals that about 50% exhibited a severe form, 32.1% a moderate form, and 14.3% a mild form (Table 1).

**Figure 1:**
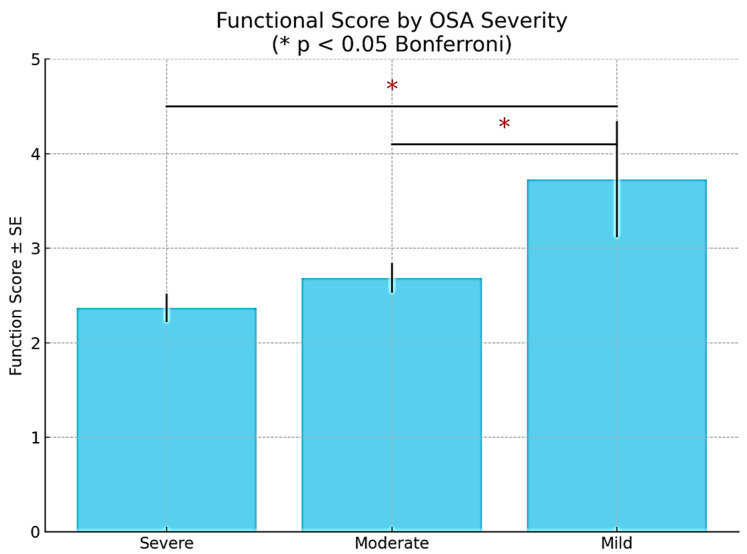
Evolution of “daily functioning” domain score in relation to OSA severity.

The analysis of the participants’ responses in the “daily activity” area indicates that obstructive sleep apnea (OSA) consistently adversely affects everyday functioning, with patients exhibiting more severe forms experiencing greater physical challenges and a reduction in energy for fundamental activities (Figure 1).

A comparative analysis of the functional score according to the severity of obstructive sleep apnea (OSA) reveals a clear trend of decreased functional capacity as the severity of the condition increases. Participants diagnosed with severe OSA recorded the lowest mean functional score (2.37 ± 0.89), followed by those with moderate OSA (2.69 ± 1.02), while the group with mild OSA exhibited the highest values (3.73 ± 1.34). Bonferroni post hoc testing showed statistically significant differences between the severe and mild OSA groups (*p* = 0.004), as well as between the moderate and mild groups (*p* = 0.045). No significant differences were found between the severe and moderate groups (*p* = 0.809). These results suggest that both moderate and severe forms of OSA negatively affect patients’ daily functioning. Thus, OSA severity is associated with a significant negative functional impact, underscoring the importance of early diagnosis and appropriate therapeutic intervention in pregnant patients affected by this condition (Figure 1).

The second domain, termed “social interactions”, assessed the challenges faced by patients diagnosed with OSA, who, in addition to their physical ailments, experienced significant difficulties in their relationships with colleagues and close acquaintances.

**Figure 2:**
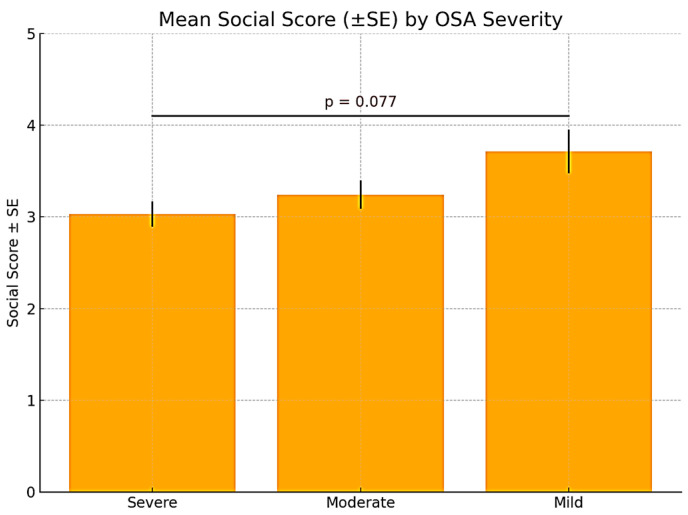
Mean social score of the “social interactions” domain in relation to OSA severity.

An upward trend was observed, with patients diagnosed with mild OSA reporting the highest average social scores (3.71 ± 1.25), followed by those with moderate OSA (3.24 ± 1.72), and the lowest scores were reported by those with severe OSA (3.03 ± 0.86). Although one-way ANOVA yielded a *p*-value close to significance (*p* = 0.076), Bonferroni post hoc analysis did not confirm statistically significant differences between the groups. The difference between the severe and mild OSA groups approached the threshold of significance (*p* = 0.077), suggesting a potential negative impact of more severe OSA on patients’ perceived social functioning (Figure 2).

The evaluation of emotional status within the “emotional status” domain revealed a substantial influence of obstructive sleep apnea (OSA) on patients’ emotional well-being, particularly in moderate and severe cases of the disorder.

**Figure 3:**
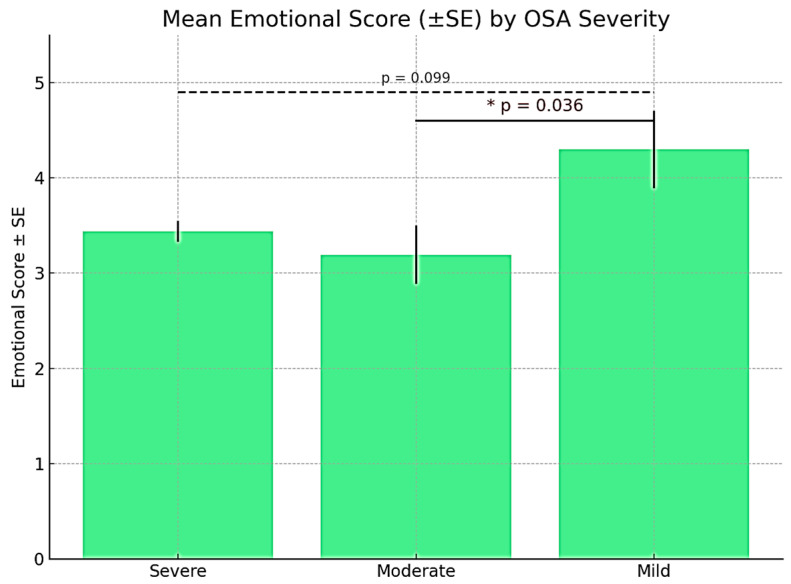
Distribution of mean values of the “emotional status” domain in relation to OSA severity.

The analysis of emotional scores according to OSA severity revealed a downward trend in emotional well-being as the severity of the disorder increased. Participants with mild OSA reported the highest emotional scores (4.30 ± 1.33), followed by those with severe OSA (3.44 ± 0.96), while participants with moderate OSA had the lowest average scores (3.19 ± 1.26). Bonferroni post hoc analysis indicated a statistically significant difference between the moderate and mild OSA groups (*p* = 0.036), suggesting that moderate OSA may significantly impair emotional well-being. Although the difference between severe and mild OSA did not reach statistical significance (*p* = 0.099), it approached the conventional threshold, indicating a potential emotional impact even in severe cases. No significant differences were observed between the moderate and severe groups (*p* = 1.000) (Figure 3).

The symptomatology of individuals with obstructive sleep apnea (OSA) is intricate and enduring, markedly affecting sleep quality and overall well-being.

**Figure 4:**
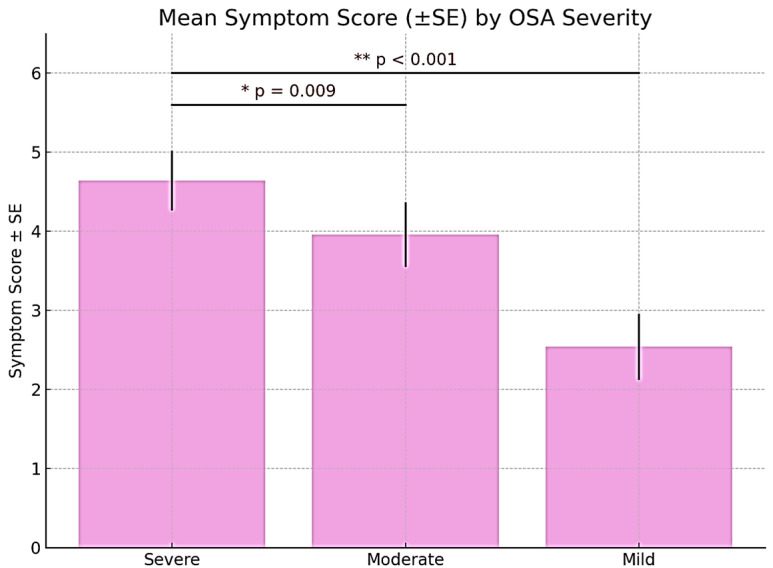
Mean values distribution of the “symptoms” domain in relation to OSA severity.

The analysis of symptom scores according to OSA severity showed a clear descending trend, with more severe forms of OSA being associated with a higher symptom burden. Participants with severe OSA recorded the highest average symptom score (4.64 ± 1.05), followed by those with moderate OSA (3.96 ± 1.42), and the lowest values were observed in the mild OSA group (2.54 ± 0.98). Bonferroni post hoc analysis revealed statistically significant differences between the severe and moderate groups (*p* = 0.009) and between the severe and mild groups (*p* < 0.001). However, the difference between moderate and mild OSA did not reach statistical significance (*p* = 0.124). These findings suggest that symptom severity is strongly correlated with the clinical stage of OSA, particularly in severe forms, highlighting the importance of early detection and management in pregnant patients experiencing sleep-disordered breathing (Figure 4).

Table 2 outlines the disparities among patients with obstructive sleep apnea (OSA) regarding mean scores across the following four critical domains: functional, social, emotional state, and symptomatology, contingent upon the severity of the illness (severe, moderate, and mild). In the functional domain, patients with severe obstructive sleep apnea (OSA) exhibited the lowest mean scores (M = 2.37), signifying considerable impairment in everyday activities, whereas those with mild OSA had a substantially higher score (M = 3.73).

The differences across the groups were statistically significant (F = 6.603, *p* = 0.005). In the social area, an increase in scores was noted with decreasing severity (from 3.03 to 3.71), however, these differences did not attain statistical significance (*p* = 0.076). Patients with severe obstructive sleep apnea (OSA) had lower emotional status scores (M = 3.44) than those with mild OSA (M = 4.30), with a statistically significant difference (*p* = 0.038), suggesting a detrimental effect of the condition on mental health. A notable disparity was evident in the symptomatology domain, where the mean score rose from 2.99 in severe OSA to 5.08 in moderate OSA, exhibiting highly statistically significant changes (F = 13.434, *p* < 0.001). This outcome verifies that the severity of obstructive sleep apnea (OSA) is directly connected with the degree of symptoms reported by patients, hence impacting their quality of sleep and life.

In the multiple regression analyses conducted across the four quality-of-life domains, age and AHI emerged as significant predictors of daily functioning (*p* = 0.001 and *p* = 0.037, respectively), suggesting that both an increasing age and OSA severity contribute to a reduced functional capacity. In the symptoms domain, BMI was the only significant factor (*p* = 0.012), indicating that a higher body mass is associated with an increased symptom burden (Table 3).

No statistically significant predictors were identified for the emotional or social domains, although sex approached significance for both daily functioning and social interaction.

## 4. Discussion

Obstructive sleep apnea (OSA) has a significant impact on quality of life, influencing multiple dimensions of daily functioning, such as physical condition, cognitive performance, emotional balance, and social relationships.

Assessing quality of life in patients with obstructive sleep apnea (OSA) is essential for understanding the overall impact of this disease on overall health and daily functioning. The SAQLI questionnaire is a validated instrument specifically designed to assess quality of life among people with OSA [22]. Recent studies have shown that the SAQLI is sensitive to changes generated by therapeutic interventions, especially continuous positive airway pressure (CPAP) treatment, making it a useful tool in monitoring the effectiveness of treatment [23,24]. This questionnaire can identify the areas of dysfunction predominantly affected, which allows for the personalization of interventions and contributes to a patient-centered approach, focused not only on symptom relief, but also on improving overall quality of life.

In our study, patients with severe obstructive sleep apnea reported significant difficulties in performing daily activities, especially in maintaining alertness, using full energy for simple tasks, and engaging in physical exercise or enjoyable activities. These results are supported by the study by Karkala et al. (2024) [25], which showed that patients with OSA present with a low level of engagement in daily activities and a general decrease in energy. Similarly, Mendelson et al. (2018) [26] found that the severity of OSA symptoms is associated with a reduced exercise capacity and limited participation in social and functional activities.

The findings of our study indicate a considerable disruption in the social life of patients with OSA, evidenced by lower mean scores in those with severe cases and significant *p*-values for factors such as “issues in partner relationships” and “rejection of family activities”. This tendency aligns with the findings of Luyster et al. (2016) [27], which emphasized that OSA impacts not only the patient, but also their life partner and familial relationships. Garbarino et al. (2020) [28] indicated a detrimental effect on social relationships and closeness, particularly in untreated or severely symptomatic instances.

Disrupted sleep, pronounced daytime drowsiness, and recurrent instances of nighttime hypoxia might disturb the brain’s neurochemical equilibrium, impairing emotional regulation and rendering patients susceptible to a precarious psychological condition [28,29]. Research indicates a notable correlation between the severity of obstructive sleep apnea (OSA) and the intensity of depression and anxiety symptoms, with a higher prevalence observed in women [30]. In addition, the impairment of emotional status often leads to a reduction in social and professional involvement, with patients frequently reporting feelings of discouragement, a lack of mental energy, and a low tolerance to daily stress [31]. This link is reciprocal: OSA not only precipitates affective problems, but the existence of sadness or anxiety can also exacerbate the adverse sense of sleep and everyday functioning.

Lower scores among patients with severe OSA in this domain (M = 3.44) indicate a significant impairment in their emotional state, especially through the frequent presence of anxiety, irritability, and feelings of depression. Our study confirms the observations in the literature that OSA is associated with an increased risk of affective disorders. Garbarino et al. (2020) and Bjornsdottir et al. (2015) showed a high prevalence of depressive and anxiety symptoms in patients with untreated OSA, highlighting the importance of an integrated psychological approach in therapeutic plans [28,32].

Obstructive sleep apnea (OSA) presents with diverse symptoms that affect both sleep quality and daytime functioning. Typical nocturnal signs include loud snoring, witnessed apneas, frequent awakenings with choking or thirst, and non-restorative sleep [32,33]. Daytime manifestations include excessive sleepiness, fatigue, cognitive difficulties, mood disturbances, and reduced libido [34,35]. Symptom severity correlates with the apnea–hypopnea index and nocturnal hypoxemia, and untreated OSA increases cardiovascular and metabolic risks [36]. Globally, OSA is underdiagnosed due to symptom normalization or misattribution, delaying treatment (Benjafield et al., 2019). Early recognition and screening are essential to prevent complications and improve quality of life [37].

Our study results show the most significant differences between severity groups in symptomatology, with higher mean scores in severe OSA (e.g., frequent awakenings, feeling of suffocation, and chronic fatigue). These data are in agreement with studies by Weaver et al. (2021) [38] and Punjabi (2020) [36], who demonstrated that symptomatology such as daytime sleepiness, extreme fatigue, and fragmented sleep is more intense in severe forms of the disease. Flemons & Reimer (1998) [13], the developers of the SAQLI, emphasized that this domain is a critical indicator of sleep quality and responds well to effective interventions such as CPAP.

Although CPAP is the standard treatment for OSA, the recent literature highlights adverse effects such as aerophagia, which is common and may impact treatment adherence and quality of life. Harding (2013) reported a 50% prevalence of CPAP-related gastrointestinal symptoms, emphasizing the need for their monitoring. In this context, the use of validated questionnaires like SAQLI is essential not only for assessing disease impact, but also for evaluating treatment-related side effects. Our findings, which reveal functional and emotional impairments, support the importance of a personalized, patient-centered approach [39].

The findings of our study regarding the negative impact of obstructive sleep apnea (OSA) on quality of life—particularly in its severe forms—are consistent with the existing literature, which emphasizes the systemic and multidimensional effects of this condition. For instance, the HypnoLaus study conducted by Heinzer et al. (2015) [40] reported a high prevalence of OSA in the general population and demonstrated a clear association between the severity of sleep-disordered breathing and a range of chronic comorbidities, such as hypertension, diabetes, and depression. These comorbidities, also frequently observed in our cohort, significantly contribute to the deterioration of patients’ quality of life. Furthermore, in line with our findings related to functional and psycho-emotional impairment, subsequent HypnoLaus publications have highlighted the need for integrated approaches that go beyond symptomatic treatment and include validated quality of life assessments, such as the SAQLI questionnaire. Thus, our data not only support trends identified in the recent literature, but also provide a novel perspective on Eastern European populations, which remain underrepresented in global research [40].

Although CPAP therapy remains the standard of care for obstructive sleep apnea (OSA), several alternative treatment options have proven effective, particularly for patients who are non-compliant or present with mild to moderate forms of the condition. Oral appliances such as mandibular advancement devices (MADs) have shown beneficial effects in reducing the apnea–hypopnea index (AHI), especially in patients with positional OSA. Fransson et al. (2022) [18] reported a 77% success rate after one year of MAD use in such patients, supporting the efficacy of this minimally invasive approach. By repositioning the mandible forward, MADs help to maintain upper airway patency during sleep, thereby alleviating symptoms and improving overall well-being [18].

Another complementary method is rapid palatal expansion (RPE), particularly indicated in children and adolescents with narrow maxillary arches and nasal obstruction. Tsolakis and Kolokitha (2023) demonstrated, through CBCT assessments, a significant increase in airway space following orthopedic treatment, which may help to reduce the risk or severity of OSA. Additionally, behavioral modifications—such as weight loss, a balanced diet, and regular physical activity—play a crucial role in improving symptoms among overweight patients. In severe cases or in the presence of structural abnormalities, surgical interventions like uvulopalatopharyngoplasty (UPPP) or maxillomandibular advancement (MMA) may be appropriate. Therefore, effective OSA management calls for an individualized, multimodal approach that integrates personalized and complementary therapies tailored to each patient’s profile [41,42].

The present study presents a series of limitations that must be taken into account when interpreting the results. First, the small sample size, consisting of only 28 participants, limits the generalizability of the conclusions and the ability to detect significant differences between subgroups. Also, the cross-sectional design of the research provides a punctual image of the impact of OSA on quality of life, without allowing for observations on its evolution over time or the effects of treatment. The absence of a control group limits the ability to draw causal inferences and generalize findings; future research should consider comparative study designs to strengthen the validity and interpretability of the results. Although participants were recruited from both Romania and Albania, we opted to pool the data due to the small sample size and the sociodemographic homogeneity of the groups. All participants were evaluated using the same inclusion criteria, diagnostic protocols, and questionnaire, minimizing cross-national variability.

The lack of a healthy control group prevents direct and rigorous comparisons of the influence of OSA with the general population. Another limitation is represented by the relative homogeneity of the participants, most of whom came from urban areas and were professionally active, which reduces the applicability of the results to other sociodemographic categories. In addition, the use of the SAQLI questionnaire, based on self-assessment, introduces a degree of subjectivity that can be influenced by emotional or contextual factors.

The lack of assessment of the impact of treatment (such as CPAP therapy) on quality of life limits the applicative value of the study in clinical practice. These limitations highlight the need for future research with larger samples, longitudinal designs, and comparative approaches to provide a broader and more valid perspective on the relationship between OSA and quality of life.

Future perspectives for obstructive sleep apnea (OSA) research include expanding sample sizes, using longitudinal designs, and integrating assessments of the impact of treatment on quality of life. Future studies should include more diverse populations and combine subjective instruments, such as the SAQLI, with objective clinical data. It is also necessary to analyze the evolution of patients before and after the initiation of therapy, especially CPAP. The development of modern screening and monitoring methods could contribute to early diagnosis and the personalization of interventions, with the aim of improving not only symptomatology, but also quality of life in the long term.

## 5. Conclusions

Obstructive sleep apnea (OSA) is a prevalent disorder that substantially affects patients’ quality of life, influencing physical functioning, mental well-being, and social interactions.

Symptoms including diurnal somnolence, lethargy, anger, and cognitive dysfunction adversely affect everyday activities and personal life. Utilizing the SAQLI questionnaire to evaluate quality of life facilitates a more precise comprehension of patient demands and enhances the efficacy of therapy adaptations. The treatment of OSA should encompass medical therapies, including CPAP therapy, alongside psychological and educational support to enhance overall health and quality of life.

## Figures and Tables

**Table 1:** Demographic characteristics of all participants.

	No	%
Age	56.54 ± 11.06 years old (minimum age 33 years old–maximum age 77 years old)	
Gender		
Female	11	39.3
Male	17	60.7
Residence		
Urban	18	64.3
Rural	10	35.7
Social status		
Employed	21	75.0
Unemployed	2	7.1
Retiree	5	17.9
OSA severity		
Severe obstructive sleep apnea	15	53.6
Moderate obstructive sleep apnea	9	32.1
Mild obstructive sleep apnea	4	14.3
AHI		
AHI > 30/h	15	53.6
AHI 16–30/h	9	32.1
AHI < 15/h	4	14.3
BMI		
Obese (>30 kg/m^2^)	12	42.9
Overweight (28–30 kg/m^2^)	11	39.3
Normal weight (<25 kg/m^2^)	5	17.9

**Table 2:** Comparative analysis of mean scores for four key domains affected by obstructive sleep apnea (OSA), according to OSA severity.

Field	OSA Severity	Mean	Std. Deviation	Minimum	Maximum	F *	Sig. *
Functional	Severe OSA	2.3700	0.58329	1.36	3.36	6.603	0.005
	Moderate OSA	2.6867	0.47429	2.00	3.36		
	Mild OSA	3.7300	1.22553	2.55	5.00		
Social	Severe OSA	3.0313	0.53954	2.23	3.77	2.866	0.076
	Moderate OSA	3.2422	0.46711	2.62	3.85		
	Mild OSA	3.7125	0.48023	3.00	4.00		
Emotional Status	Severe OSA	3.4380	0.43486	2.55	3.82	3.753	0.038
	Moderate OSA	3.1933	0.92480	2.00	4.82		
	Mild OSA	4.2975	0.80946	3.09	4.82		
Symptoms	Severe OSA	2.9887	0.79750	1.96	4.22	13.434	0.000
	Moderate OSA	4.0744	0.69757	3.22	5.74		
	Mild OSA	5.0850	0.90850	3.74	5.74		

* Anova test.

**Table 3:** Coefficients of multiple linear regression models predicting SAQLI domain scores by sex, age, AHI, and BMI.

Model		Unstandardized Coefficients		Standardized Coefficients	t	Sig.
		B	Std. Error	Beta		
Daily function domain (constant)		1.052	0.284		3.703	0.001
	Sex	−0.408	0.223	−0.256	−1.827	0.081
	Age	1.056	0.271	0.557	3.901	0.001
	AHI	0.459	0.207	0.428	2.215	0.037
	BMI	0.168	0.196	0.159	0.855	0.401
Social interaction domain (constant)		2.309	0.248		9.294	0.000
	Sex	0.351	0.195	0.321	1.801	0.085
	Age	0.023	0.237	0.018	0.098	0.923
	AHI	0.197	0.181	0.266	1.085	0.289
	BMI	0.194	0.172	0.268	1.129	0.270
Emotional status domain (constant)		2.541	0.385		6.606	0.000
	Sex	−0.318	0.302	−0.213	−1.053	0.303
	Age	0.486	0.366	0.273	1.326	0.198
	AHI	0.401	0.281	0.398	1.430	0.166
	BMI	0.062	0.266	0.062	0.231	0.819
Symptoms domain (constant)		1.577	0.423		3.726	0.001
	Sex	0.039	0.332	0.018	0.117	0.908
	Age	0.059	0.403	0.023	0.147	0.884
	AHI	0.368	0.309	0.250	1.191	0.246
	BMI	0.799	0.293	0.554	2.731	0.012

## Data Availability

The original contributions presented in this study are included in the article. Further inquiries can be directed to the corresponding author.
